# Two vicious circles associated with the aging of the immune system in the development of severe forms of COVID-19

**DOI:** 10.3389/fragi.2023.1260053

**Published:** 2023-09-14

**Authors:** Miodrag Vrbic, Ana Milinkovic

**Affiliations:** ^1^ Clinic for Infectious Diseases—UCC Nis, Nis, Serbia; ^2^ Chelsea and Westminster Foundation Trust and Imperial College London, London, United Kingdom

**Keywords:** COVID-19, MODS, senescence of adoptive immunity, sepsis, pathogenesis

## Abstract

**Background:** The immune-inflammatory response is the basis of the pathophysiology of SARS-Cov-2 infection. In severe cases of COVID-19 uncontrolled systemic inflammatory response causes multiorgan dysfunction (MODS), as the most common immediate cause of death. Unfavorable outcome of the COVID-19 most often occurs in elderly patients. The aim of the study was to establish parameters with prognostic significance in severe cases of COVID-19 according to life years, laboratory markers of sepsis and MODS, as well as the number of peripheral CD4^+^ and CD8^+^T lymphocytes in 20 consecutively selected critically ill patients.

**Results:** Eleven subjects were male, 9 female, mean age 73.45 ± 11.59, among which the oldest patient was 94 and the youngest 43 years. All the patients met the sepsis and MODS criteria. Increased age and low CD4^+^ and CD8^+^T cell counts were identified as independent predictors of death. Only the two youngest patients (43 and 50 years old) survived 28 days, and they are the only ones with a CD4 lymphocyte count above 500 cells/mm^3^.

**Conclusion:** Senescence of the immune system is mostly characterized by reduced regenerative capacity of adaptive immunity with diminished ability to respond to new antigens and a manifested proinflammatory phenotype. Additional reduction of protective capacity by further deterioration of T cell quantity and quality due to sepsis itself and mutual interaction of senescent T cells and vascular endothelial cells in the induction of cytokine storm represent two complementary vicious cycles in the development of sepsis-related multiorgan dysfunction.

## Introduction

Today, much is known about the clinical course and mortality of SARS-CoV-2 infection. Furthermore, current findings emphasize that the immune-inflammatory response plays a key role in its pathophysiology. Considering the wide distribution of angiotensin-converting enzyme 2 (ACE2), the functional receptor through which SARS-CoV-2 enters host cells, COVID-19 is a systemic disease. The first step in understanding of its pathogenesis is the abundant expression of ACE2 on respiratory tract epithelial cells and vascular endothelial cells (Pons et al., 2020). They represent an important component of innate immunity, causing a proinflammatory reaction, particularly violent in the presence of SARS-CoV-2 viremia, that is not detectable in mild and asymptomatic cases of COVID-19 (Richter et al., 2021). In severe cases of COVID-19, systemic inflammatory hyperactivation in the form of a cytokine storm causes sepsis-related MODS as the most common immediate cause of death (De Roquetaillade et al., 2021; Elezkurtaj et al., 2021).

Adverse disease outcomes are far more common in older patients (Santesmasses et al., 2020). The aging process itself is accompanied and guided by the aging of the immune system, primarily senescence of adoptive immunity, which is characterized by a reduction of its regenerative capacity, with a reduced protective capacity and a manifested pro-inflammatory phenotype. (Anna et al., 2019; Cunha et al., 2020). Dealing with these circumstances could contribute to a better understanding of the pathophysiological role of adaptive immunity, which is still not fully explained, in the development of severe forms of COVID-19.

In accordance with the given assumptions, the aim of the study was to establish the years of life, presence of sepsis and MODS, values of additional factors with confirmed prognostic significance and number of peripheral CD4^+^ and CD8^+^T lymphocytes in critically ill patients with COVID-19.

## Methods

The study was conducted at the Clinic for Infectious Disease of University Clinical Center Nis, Serbia, from January to March 2022. Twenty patients were consecutively selected at the beginning of invasive mechanical ventilation, during hospitalization due to COVID-19.

Presence of mechanical ventilation, frequent need for sedation and use of vasoactive agents made impossible for us to use the clinical criteria (such as SOFA or qSOFA scoring) in the assessment of the presence and severity of sepsis and MODS. Therefore, various laboratory markers of sepsis and MODS were determined, including: interleukin 6 (IL-6), procalcitonin (PCT), C-reactive protein (CRP), white blood cell count (WBC), lymphocyte %, platelets count (PLT), glycemia, creatinine, lactate, international normalized ratio (INR), and PaO2/FiO2. Additionally, the values of lactate dehydrogenase (LDH), ferritin and D-dimer, with a confirmed prognostic significance for COVID-19, were determined. The numbers of peripheral CD4^+^ and CD8^+^T lymphocytes were determined by flow cytometry (BD FACSCount™ Reagent Kit).

Analyzes were performed on blood samples taken on the same day, during the patients' stay in the intensive care unit. The results were presented as arithmetic means and standard deviations, with assessment of their association with the probability of death by univariate and multivariate Cox regression analysis and with Kaplan-Meier curve of 28 days survival.

## Results

All the patients met the sepsis and MODS criteria. Also, increased values of LDH, ferritin and D-dimer, as well as depletion of CD4 + T and CD8 + T lymphocytes were recorded (Table 1).

**TABLE 1:** Laboratory results presented as arithmetic means and standard deviations.

Analysis	Values	Reference ranges
IL-6	304.89 ± 235.75 pg/mL	<7 pg/mL
PCT	27.66 ± 25.39 ng/mL	0.0–0.05 ng/mL
CRP	134.24 ± 115.56 mg/L	0.0–5.0 mg/L
WBC count	16.4 ± 9.4 × 10^9^/L	4.5 to 11.0 × 10^9^/L
Lymphocyte %	5.3 ± 2.86%	20%–40%
PLT	118 ± 74 × 10^9^/L	120.0–380.0 × 10^9^/L
Glycemia	14.0 ± 5.3 mmol/L	3.9–6.1 mmol/L
Creatinine	240.2 ± 187.4 μmol/L	53.0–115.0 μmol/L
Lactate	5.0 ± 2.4 mmol/L	<2.0 mmol/L
INR	1.00 ± 0.16	0.80–1.20
PaO2/FiO2	115 ± 27	≥400
LDH	553 ± 354 U/L	220.0–450.0 U/L
Ferritin	849.2 ± 389.3 μg/L	20.0–250.0 μg/L
D-dimer	5,939 ± 6,464 ng/mL	0.0–250.0 ng/mL
CD4^+^ T cells	172 ± 167 cells/mm^3^	500 to 1,500 cells/mm^3^
CD8^+^ T cells	147 ± 191 cells/mm^3^	50 to 1,000 cells/mm^3^

By univariate regression analysis, increased age and low CD4^+^T and CD8^+^T cells counts were identified as independent predictors of death. In the multivariate model of Cox regression analysis, containing the 3 given variables, only age retained statistical significance as the factor associated with mortality, probably as a cumulative expression of changes in the number of CD4^+^T and CD8^+^T lymphocyte cells (Table 2).

**TABLE 2:** Association with the probability of death by univariate and multivariate Cox regression analysis.

Univariate cox regression analysis				
Factor	OR	95% CI limits for OR		*p*
		LL	UL	
**Age**	**1.108**	**1.031**	**1.190**	**0.005**
**Gender**	0.971	0.382	2.472	0.951
**IL-6**	0.996	0.990	1.003	0.288
**PCT**	1.006	0.990	1.022	0.471
**CRP**	1.003	0.999	1.007	0.193
**WBC**	0.989	0.930	1.050	0.710
**Lymphocyte %**	0.902	0.755	1.078	0.256
**PLT**	0.996	0.990	1.003	0.288
**Glycemia**	1.010	0.906	1.125	0.860
**Creatinine**	1.001	0.998	1.005	0.451
**Lactate**	1.049	0.890	1.236	0.569
**INR**	0.914	0.609	1.371	0.664
**PaO2/FiO2**	0.989	0.966	1.013	0.354
**LDH**	1.000	0.998	1.001	0.590
**Ferritin**	1.001	1.000	1.002	0.149
**D-dimer**	1.000	1.000	1.000	0.903
**CD4^+^ T cells**	**0.996**	**0.992**	**0.999**	**0.022**
**CD8^+^ T cells**	**0.995**	**0.990**	**0.999**	**0.028**
Multivariate Cox regression analysis				
**Age**	**1.119**	**1.020**	**1.227**	**0.017**
**CD4^+^ T cells**	1.005	0.996	1.013	0.292
**CD8^+^ T cells**	0.994	0.986	1.013	0.165

**FIGURE 1:**
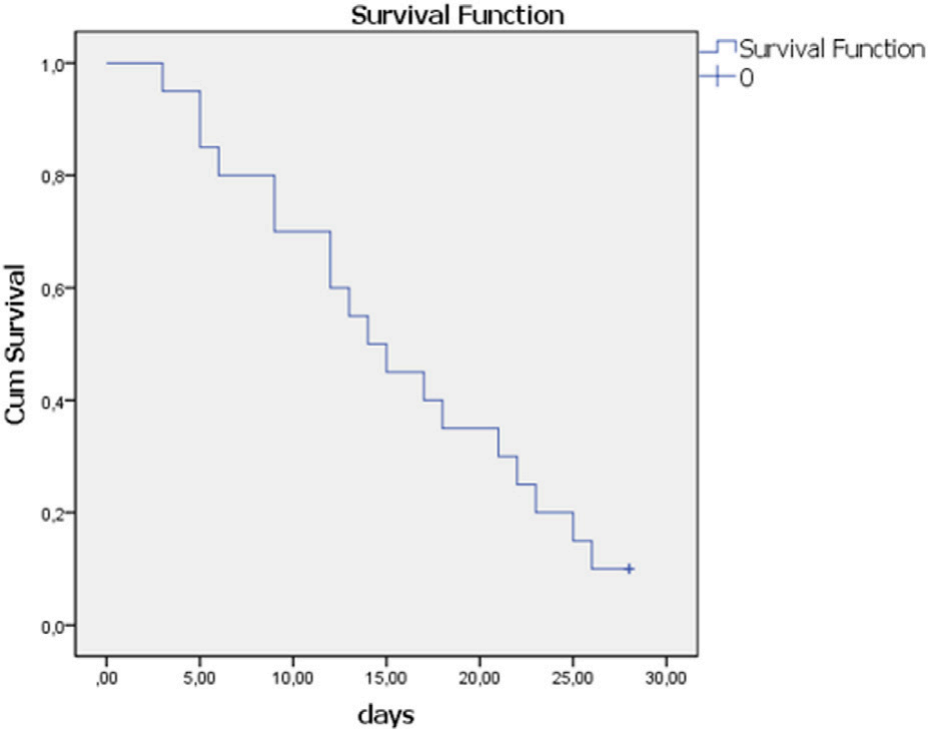
Kalpana-Meier curve of 28-day survival average survival time was 15.55 + 1.75 days.

Only the two youngest patients (43 and 50 years old) survived 28 days, and these were the only ones with a CD4^+^T lymphocyte count above 500 cells/mm^3^ (Figure 1).

## Discussion

Before the COVID-19 pandemic, in a cohort of patients with acute hypoxemic respiratory failure death was rarely due to refractory pulmonary dysfunction. In SARS-CoV-2 infection, pulmonary dysfunction is present in the vast majority of cases at the time of death, as the most conspicuous sign of MODS (Ketcham et al., 2021).

The development of severe forms and mortality from COVID 19 are associated with age-related changes in the immune response (Bartle et al., 2021). The pathogenetic substrate of MODS development is the cytokine storm primarily triggered by vascular endothelial damage (Otifi and Adiga, 2022). Further, as a conditional part of the acquired immunity, endothelial cells represent the interface between the innate and adaptive immune responses (Schleimer et al., 2007). They are important for the recruitment and activation of T-cells, which with aging exhibit increased production of proinflammatory cytokines (Fukushima et al., 2018; Degauque et al., 2021). At the same time, the senescent T cells may contribute to the immunopathological mechanism of additional endothelial damage, in the vicious circle of inflammatory hyperactivation (Covre et al., 2020; Zhang et al., 2021). Clinically, the significant role of adaptive immunity is indicated by the time required for its activation, 2 weeks on average, considering that, in addition to the incubation period, serious forms of the disease usually develop 10 days after the appearance of the first symptoms (Blair et al., 2021).

Another vicious circle present is a deterioration of the quantity and quality of T cells, that generally characterizes sepsis, which results in an additional reduction of the protective capacity of adaptive immunity, already weakened by immunosenescence (Cabrera-Perez et al., 2014; Danahy et al., 2016). From there, the presence of SARS-CoV-2 viremia and its duration were directly correlated with the degree of inflammation and mortality (Hagman et al., 2022), while depletion rate of peripheral CD8 + T cells reflected the severity of the disease, and reduced CD4 +T cell count was independently associated with increased in-hospital mortality in patients with COVID-19 (Wen et al., 2021).

## Conclusion

The development of severe forms and mortality from COVID-19, as well as sepsis in general, is associated with an overwhelming life-threatening immune response associated with aging.

Driven primarily by changes of adaptive immunity, this process is mostly genetically programmed, which greatly reduces the possibility of therapeutic interventions.

From there, the most promising are therapeutic interventions which reduce the possibility of excessive immune activation, such as the administration of virustatic agents and neutralizing monoclonal antibodies early in the course of infection.

## Data Availability

The original contributions presented in the study are included in the article/Supplementary material, further inquiries can be directed to the corresponding author.
